# Evolutionary biologic changes of gut microbiota in an ‘adenoma-carcinoma sequence’ mouse colorectal cancer model induced by 1, 2-Dimethylhydrazine

**DOI:** 10.18632/oncotarget.13443

**Published:** 2016-11-18

**Authors:** Teng Sun, Shanglong Liu, Yanbing Zhou, Zengwu Yao, Dongfeng Zhang, Shougen Cao, Zhiliang Wei, Bin Tan, Yi Li, Zheng Lian, Song Wang

**Affiliations:** ^1^ Department of General Surgery, Affiliated Hospital of Qingdao University, Qingdao, China; ^2^ Department of General Surgery, Qingdao municipal hospital, Qingdao, China; ^3^ Department of General Surgery, Yantai Yuhuangding Hospital, Yantai, China; ^4^ Department of Epidemiology and Health Statistics, Qingdao University Medical College, Qingdao, China; ^5^ Department of General Surgery, Zhucheng People's Hospital, Weifang, China; ^6^ Department of General Surgery, Linzi District People's Hospital, Zibo, China

**Keywords:** colorectal cancer, microbiology, animal model

## Abstract

The molecular biological mechanisms underlying the evolutionary biologic changes leading to carcinogenesis remain unclear. The main objective of our study was to explore the evolution of the microbiota community and molecules related with CRC in the dynamic transition from normal colon epithelium to premalignant adenoma with the aid of an ‘adenoma–carcinoma sequence’ mouse CRC model induced by DMH. We generated a modified mouse CRC model induced by DMH for DNA sequences, and characterized the molecular networks. Data from 454 pyrosequencing of the V3- V5 region of the 16S rDNA gene and immunohistochemical detection of *APC*, *P53*, *K-RAS* and *BRAF* genes were assessed with Principal coordinates, UniFrac, and Kruskal-Wallis rank sum test. The inflammatory group showed enrichment of *Bacteroidetes* and *Porphyromonadaceae* (*P* < 0.01). OTUs affiliated with *Firmicutes* were enriched in the hyperproliferative group (*P* < 0.01). *Rikenellaceae* and *Ruminococcaceae* showed an increasing trend during the CRC process while the opposite pattern was observed for *Prevotellaceae*and *Enterobacteriaceae*. OTUs related to *Alistipes finegoldii* were significantly increased during CRC development, *P53*, *K-RAS* and *BRAF*, were gradually increased (*P* < 0.05). Conversely, expression of *APC* was decreased during the course of development of CRC. Our results demonstrate that the biological evolutionary shift of gut microbiota, characterized by a gradual decrease in ‘driver’ bacteria and an increase in DNA damage-causing bacteria, is accompanied by tumor development in the CRC model. The synergistic actions of microbiota dysbiosis and effects of bacterial metabolites on related molecular events are proposed to contribute to the progression of CRC tumorigenesis.

## INTRODUCTION

Colorectal cancer is the third most common cancer type in men and the second most common in women, and reported as the fourth leading cause of cancer-related mortality worldwide [1, 2]. The issue of whether the poor prognosis and high mortality of CRC are partially attributed to pathogenesis remains unclear. The well-known ‘adenoma-carcinoma sequence’ is known to play a significant role in CRC development [3, 4]. The molecular genetic basis posits that accumulating somatic and germ-line mutations drive epithelial dysplasia and hyperproliferation in the colon, ultimately causing CRC [3, 5]. The most commonly mutated genes include tumor suppressors (*APC*, *P53* and the *β-catenin* gene) and oncogenes (*K-RAS*, *BRAF* and *MYC*). The triggers for these mutations are multifactorial in origin, associated with infectious agents and high exposure of tissues to microbiota, but remain elusive in many cases [6]. From this perspective, Tjalsmaet *et al*. [7] proposed a bacterial driver-passenger model explaining microbiota community involvement during CRC development, which may contribute to the genetic paradigm of the ‘adenoma-carcinoma sequence’. The group suggested that the colonic mucosa is colonized by bacterial drivers, defined as intestinal bacteria with procarcinogenic features that initiate CRC development. Bacterial drivers contribute to the initiation of pre-malignant lesions and accumulation of a series of mutations during the adenoma–carcinoma sequence, causing persistent inflammation, increased cell proliferation and production of genotoxic substances. With changes in the tumor microenvironment, pathogenic bacterial drivers are gradually replaced by passenger bacteria with a competitive advantage in the tumor niche. The human colon harbors as many as 36,000 bacterial species and over 100 trillion aerobic and anaerobic bacteria [8, 9], and has been identified as the anatomical location with the highest abundance of microbes [10]. The number of bacterial cells in the gut exceeds all other eukaryotic cells in the human body by a factor of 10 [11, 12]. However, imbalance or microbiota dysbiosis in the gut may induce inflammation or intestinal barrier dysfunction. In addition, existing bacteria may also enhance the susceptibility to disease, such as *Helicobacter pylori* to gastric cancer and *human papilloma virus* to cervical cancer.

Previous studies demonstrating an association of one or more microbial species with CRC have implied that gut microbiota may be a driver of CRC tumorigenesis and not connected with colitis [10]. Moreover, the differences in microbial species between CRC tumor and control tissues have been compared. However, no studies to date have successfully identified the exact bacterial strain that causes colorectal cancer. A number of researchers have reported significantly higher abundance of *Fusobacterium* species colorectal adenomas, compared to controls. For instance, Kostic and colleagues [13–16] revealed enrichment of *Fusobacterium* species in CRC, relative to adjacent normal tissue. Subsequently, the group reported that *Fusobacterium nucleatum* enhances intestinal tumorigenesis and modulates the tumor immune microenvironment. A recent study showed a longitudinal shift in the microbial community and molecular networks with colitis-associated CRC, demonstrating that phylotype shifts during this process are complex and highly dynamic [17]. However, the role played by gut microbiota in ‘adenoma-carcinoma sequence’ CRC pathogenesis is yet to be established. In particular, evidence of evolutionary microbiota alterations is scarce. Further studies are therefore necessary to uncover the role of microbiota in the evolutionary process from colorectal atypical hyperplasia to adenoma. The development of next-generation sequencing technologies has allowed the analysis of fecal microbiota at a level of detail that was previously not achievable. The aim of the current study was to investigate the evolutionary biologic changes of the gut microbiota in tumor progression from normal colon epithelium to premalignant adenoma and subsequently invasive adenocarcinoma, with a view to establishing the potential roles of different gut microbes in the specific molecular events characterizing transition to adenocarcinoma in an ‘adenoma–carcinoma sequence’ mouse CRC model.

## RESULTS

### Progression of the ‘adenoma-carcinoma sequence’ in a mouse model

**Figure 1:**
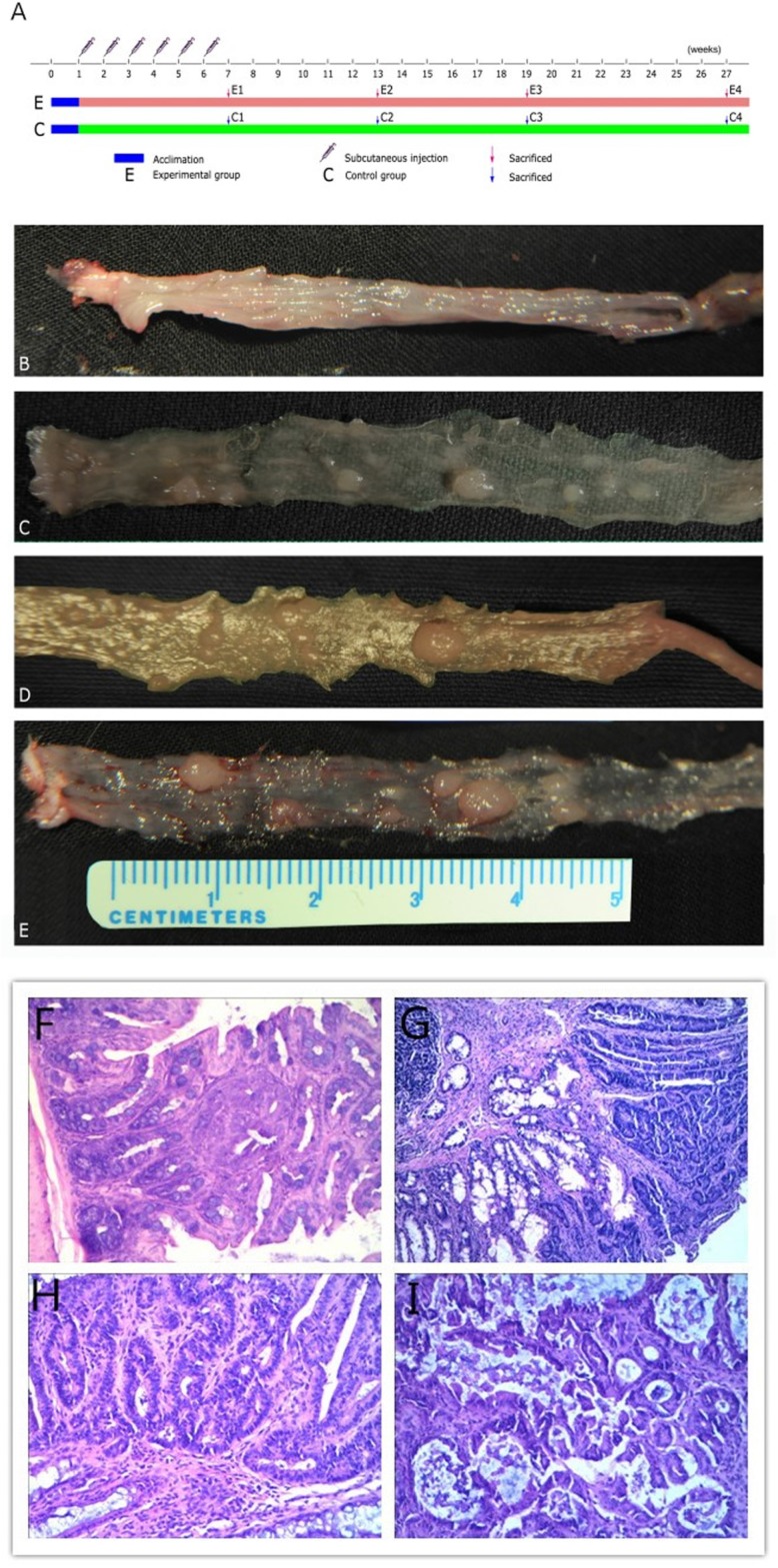
Experimental protocol, representative images of experimental groups and photomicrographs showing pathological characteristics (**A**) Experimental procedures. (**B**) Inflammation group. (**C**) Hyperproliferation group. (**D**) Adenoma group. (**E**) Adenocarcinoma group. Representative photomicrographs showing (**F**) mucosalinflammation, (**G**) hyperproliferation tissue, (**H**) adenoma and (**I**) adenocarcinoma magnified 20×.

According to the experimental protocol (Figure 1A), no obvious macroscopic lesions were observed in colon mucosa on week 6 after the first drug injection (Figure 1B), but deeper staining for colon epithelial cell nucleus and atypical hyperplasia upon microscopic examination were observed (Figure 1F). Polyps were first observed on week 12 (Figure 1C, 1G). However, the majority of adenomas were detected on week 18 (Figure 1D, 1H). Unfortunately, 42 mice died due to cachexia. The size and numbers of polyps increased with time. On week 12, only one mouse contained polyps 3 mm diameter in the colon, while on week18, enlarged polyps with diameters of 5 mm were observed (Figure 1E). In the last stage (week 26), the majority of mice (17/20) of the model group had developed polyps, some of which showed integration. Pathological examination disclosed adenocarcinoma (Figure 1I).

### Overview of 454 pyrosequencing

After pyrosequencing on the Roche 454 FLX+ platform, a total of 1,080,304 raw reads were generated for all 151 samples. Following sample date split and read filter, 559,286 effective reads were generated with an average of 3,703 high-quality sequences per sample. The total number of OTUs at 97% identity was 5,689, with an average of 226 OTUs per sample. The observed species index was used to estimate microbial richness, and the Shannon index used to assess the diversity and evenness of gut microbiota in each sample. We have already observed the plateau of the refraction curve and the curve to flatten with current sequencing, suggesting that the Shannon and observed species indices estimated for all samples reached stable values at this sequencing depth. From the observed species indices, statistically significant differences were determined between each group (*P* = 0.00002).

### Structure and diversity variations of fecal microbial communities between control and experimental groups

**Figure 2:**
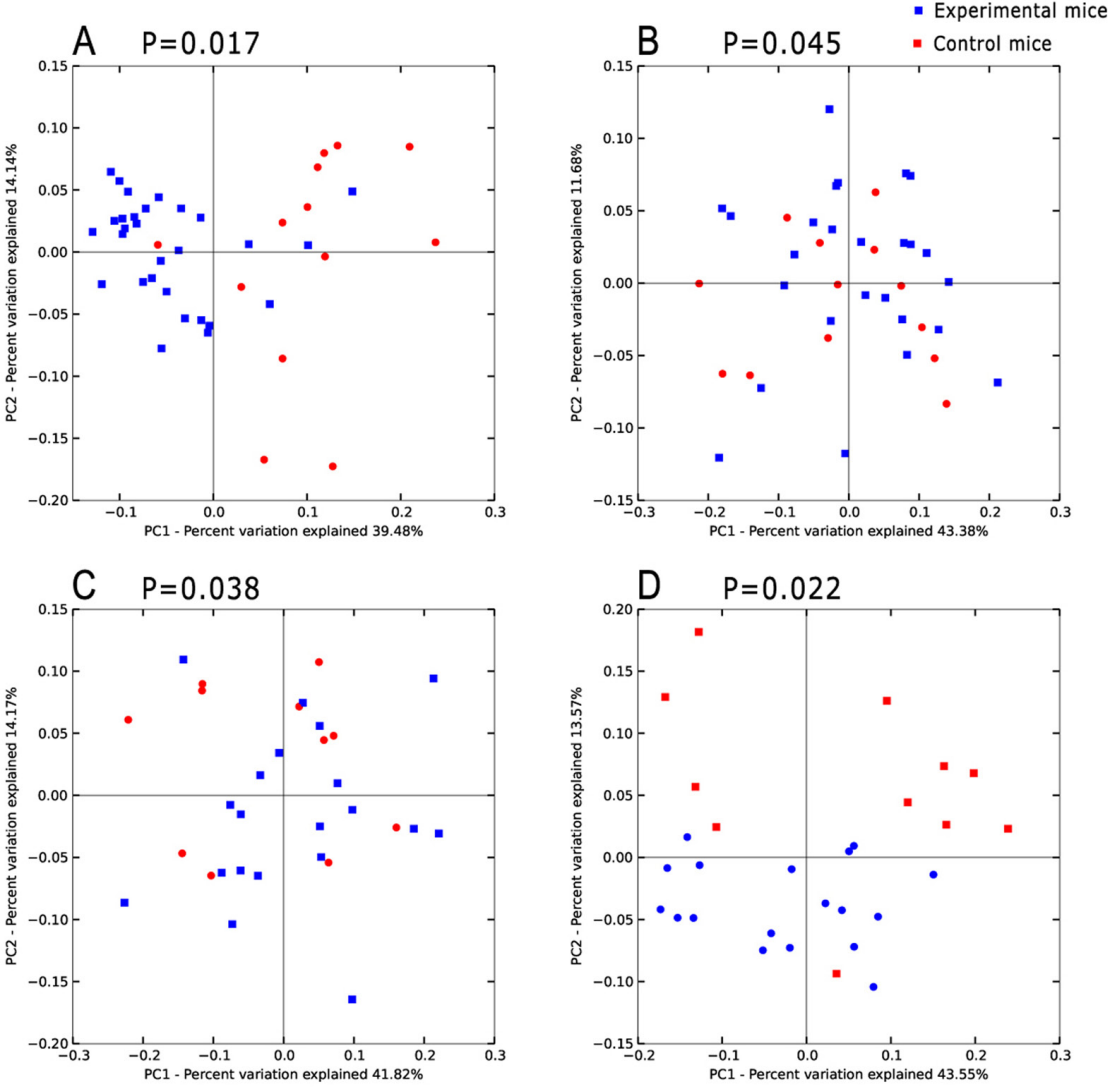
Principal coordinates analysis(PCoA)scores plot basedon relative abundance of OTUs (97% similarity level) (**A**–**D**) represent inflammatory, hyperproliferation, adenoma and carcinoma groups and the relevant control group respectively. Each symbol represents a sample. The blue symbol represents experimental mice and the red symbol control mice. Principal coordinates analysis was carried out using QIIME v1.5.0.

For beta diversity analysis, Principal Coordinates Analysis (PCoA) based on weighted UniFrac metric was employed to obtain an overview of the microbiota communities of the control and experimental groups of mice. The data revealed significant separation between mice with epithelium dysplasia, those developing cancer and healthy controls (Figure 2).

**Figure 3:**
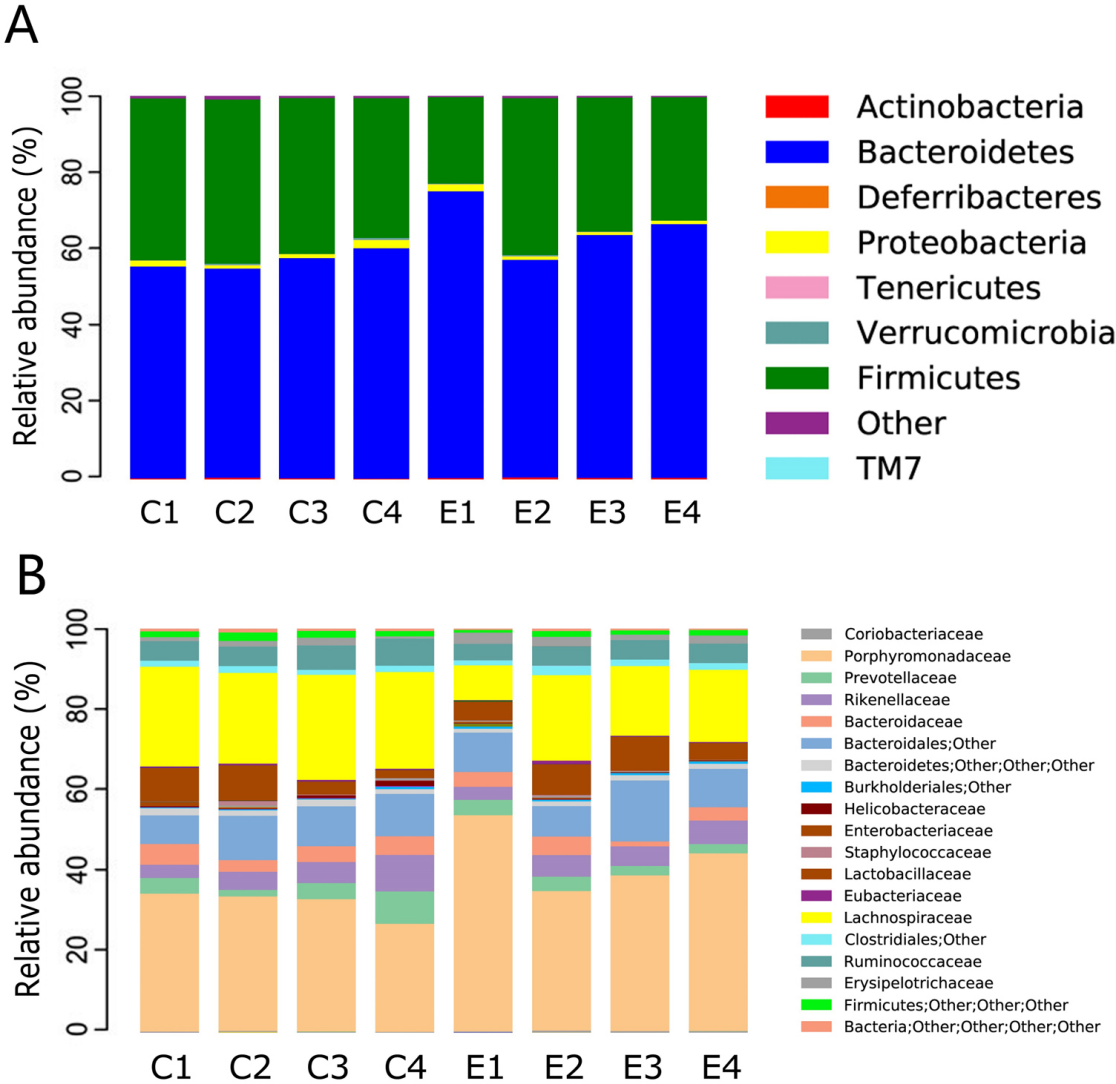
Relative abundance of the dominant phyla and family of each group ‘C’ representsthe control group, ‘E’ the experimental group. Significant difference analyses were calculated using Metastats (http://metastats.cbcb.umd.edu/), and *P* values corrected in R (Version 2.15.3).

At the phylum level, eight phyla were present in all samples, the predominant being *Bacteroidetes* and *Firmicutes*. The relative abundance of the two phyla was > 90% (Figure 3A). The relative abundance of *Bacteroidetes* species was 56.3% (54.5–60.1%) and 64.8% (63.3–74.8%) in the control and experimental groups, while that of *Firmicutes* was 41.5% (36.4–42.8%) and 33.6% (22.5–40.9%), respectively. The remaining phyla were *Verrucomicrobia, Actinobacteria, Proteobacteria, Tenericutes, Deferribacteres,* and *TM7*. Notably, *Bacteroidetes* was more highly enriched in the gut microbiota of experimental groups than control groups with statistically significant differences (77.65% vs. 56.72%, *P* = 0.001), whereas Firmicutes was markedly less abundant in the experimental groups (19.44% vs. 40.81%, *P* = 0.001). Diversity changes were additionally examined. *Deferribacteres* was absent in the hyperproliferation, adenoma and adenocarcinoma groups, with significant differences from the control group (0.01% vs. 0%, *P* = 0.010). However, no significant changes were evident with the other phyla.

At the family level, the predominant families in the control group were *Porphyromonadaceae* and *Lachnospiraceae* (Figure 3B). Families with relative abundance of > 1% in the control group were *Rikenellaceae*, *Prevotellaceae*, *Ruminococcaceae*, *Bacteroidaceae*, *Lactobacillaceae, Helicobacteraceae*, *Erysipelotrichaceae*, and *Staphylococcaceae*. The gut microbial community was clearly altered during intestinal tumorigenesis. The predominant families were still *Porphyromonadaceae* and *Lachnospiraceae*, but the remaining families with relative abundance > 1% were *Lactobacillaceae*, *Prevotellaceae*, *Bacteroidaceae, Rikenellaceae*, *Erysipelotrichaceae* and *Ruminococcaceae*. *Porphyromonadaceae* (56.01% vs. 35.06%, *P*= 0.001, FDR = 0.015), *Clostridiaceae* (0.08% vs. 0%, *P* = 0.003, FDR = 0.022), *Peptostreptococcaceae* (0.04% vs. 0%, *P* = 0.003, FDR = 0.017), *Prevotellaceae* (3.92% vs. 1.65%, *P* = 0.018, FDR = 0.066), *Coriobacteriaceae* (0.37% vs. 0.19%, *P* = 0.014, FDR = 0.092), and *Lactobacillaceae* (7.81% vs. 3.47%, *P* = 0.011, FDR = 0.064) were enriched in the experimental groups whereas *Lachnospiraceae* (23.31% vs. 6.90%, *P* = 0.003, FDR = 0.022), *Ruminococcaceae* (6.95% vs. 4.56%, *P* = 0.033, FDR = 0.111), and *Bacteroidaceae* (4.04% vs. 1.16%, *P* = 0.002, FDR = 0.035) were enriched in the control groups. Interestingly, in our study, *Clostridiaceae*, *Peptostreptococcaceae* and *Sutterellaceae* were only found in the experimental groups. However, *Dermabacteraceae, Flavobacteriaceae, Brucellaceae, Caulobacteraceae, Mycoplasmataceae*, and *Deferribacteraceae* were depleted upon CRC development. Although these families exhibited low abundance, variations were statistically significant.

### Evolutionary biologic changes and identification of key phylotypes during the process of CRC tumorigenesis

**Figure 4:**
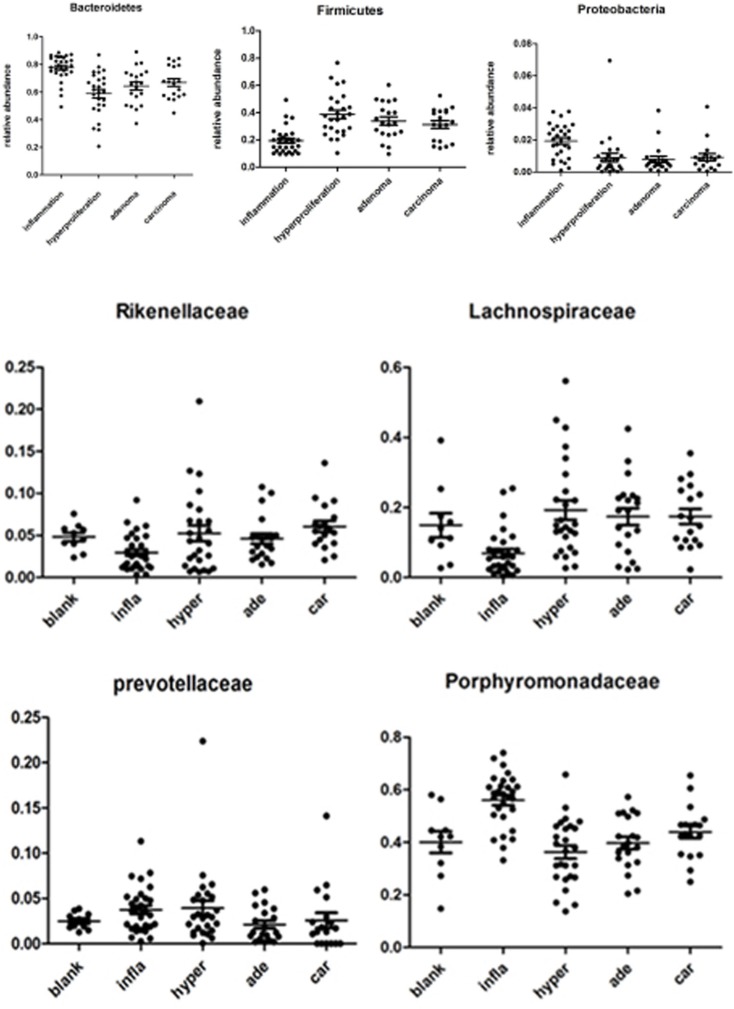
Relative abundance of the dominant phyla and families in samples of experimental groups The Kruskal-Wallis rank sum test was employed to evaluate the differences between each group.to P0 was color scaled from red (highest) to black (middle) to green (lowest). Grey indicates that no relevant expression was detected.

As specified previously, microbiota structure and diversity were obviously changed during carcinogenesis, although the specific phylotypes causing these variations and dynamic patterns of gut microbiota are yet to be identified. Time-dependent analysis of the microbiota community during tumorigenesis with the Kruskal-Wallis rank sum test revealed that at the phylum level, *Bacteroidetes* was significantly enriched in the inflammatory group (*P* < 0.01). However, in the hyperprolifarative, adenoma and adenocarcinoma groups, *Bacteroidetes* displayed decreased abundance. *Firmicutes* showed lower abundance in the inflammatory group (*P* < 0.01), but was significantly increased in the hyperprolifarative group (*P* < 0.01). *Proteobacteria* was the third most abundant phylum in the model groups, and interestingly, was also enriched in the inflammatory group (*P* < 0.01) but decreased gradually during the course of tumor development(Figure 4A).

At the family level, we analyzed the microbial composition of each group. The most abundant family was identified as *Porphyromonadaceae*, whereas the individual composition was variable, accounting for 13.71–74.03%. *Lachnospiraceae* was the second most abundant family in the model groups, ranging from 0.37% to 56.06%. In addition, *Porphyromonadaceae* and *Prevotellaceae* were enriched in the inflammatory group, but showed a significant decrease in the hyperprolifaration and adenoma groups (*P* < 0.01). Conversely, *Lachnospiraceae* was enriched in the hyperproliferation, adenoma and adenocarcinoma groups (*P* < 0.01). *Rikenellaceae* and *Ruminococcaceae* were increased during tumorigenesis. Interestingly, *Bacteroidaceae* and *Enterobacteriaceae* were enriched at the early stage, but decreased from the interim of tumor formation (Figure 4B).

**Figure 5:**
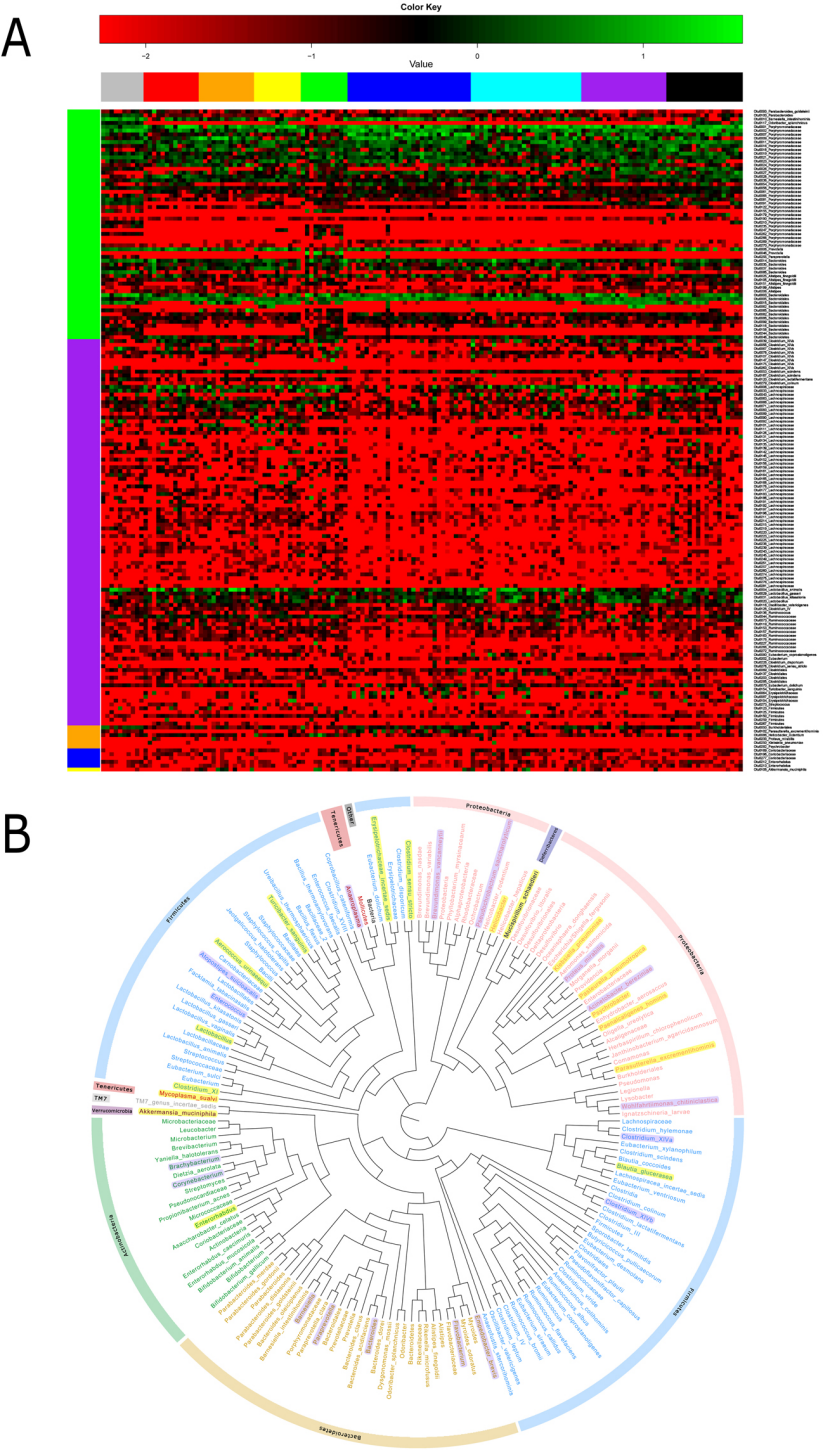
Heatmap and phylogenetic tree of significant differential OTUs (**A**) Log-fold-change and heatmap of significant differential OTUs (P < 0.05). (**B**) Significant differential genera between the experimental and control groups. Generashaded in yellow represent enrichment in the experimental group, and thoseshaded in purple shade represent enrichment in the control group. The analysis was by QIIME (Version 1.50) software, using an iterative algorithm to obtain clustering tree to R (Version 2.15.3) drawing. Clustering method UPGMA (Unweighted Pair Group Method with Arithmetic mean).

To further detect the phylotypes of microbial that contributed to longitudinal microbiota community changes, analysis based on 97% similarity OTU was performed. After filtering OTUs with very low reads, 287 OTUs with97% similarity remained (Figure 5). In an extract test, 19 OTUs were found to be significantly different between the model and control groups (*P* < 0.05). Among these, 12 were higher and 7 were lower in the model group. Two OTUs were affiliated with the species *Alistipes finegoldii* (*P* < 0.01) and three OTUs belonging to the genus *Clostridium* (*P* < 0.05) were significantly higher in the model group. An OTU related to the species *Lactobacillus animals* (*P* < 0.05) was significantly enriched in the control group and decreased with evolution of the adenoma–carcinoma sequence. In terms of structural changes, six OTUs affiliated with *Parasutterella excrementihominis, Akkermansia muciniphila, Odoribacter splanchnicus, Turicibacter sanguinis, Clostridium disporicum* and *Klebsiellapneumoniae* were only shown in the model group and two OTUs related to *Odoribacter splanchnicus* and *Proteusmirabilis* were depleted in the model group. We additionally investigated OTU variations during the ‘adenoma-carcinoma’ sequence. The results showed that OTUs belonging to *Clostridium lactatifermentans (OTU0201)* and *Bacteroidesdorei (OTU0051)* were significantly different between the hyperproliferation, adenoma and carcinoma groups. However, abundance in the adenoma group was lower. Conversely, three OTUs related to *Alistipes finegoldii (OTU0151,0105,0022)* showed significant enrichment in the adenoma group, and abundance was consistently increased during tumor development.

### Microflora variation at the later period of tumor formation

**Figure 6:**
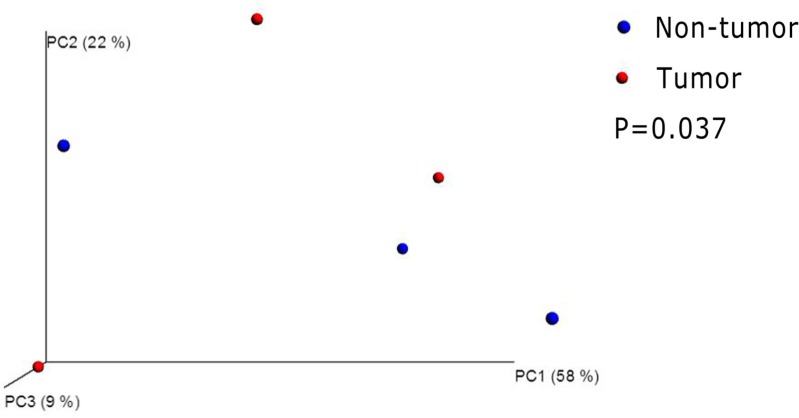
Principal coordinates analysis(PCoA)scores plot basedon relative abundance of OTUs (97% similarity level) Each symbol represents a sample. The blue symbol represents non-tumor mice and the red symbol represents tumor mice. Principal coordinates analysis were carried out using QIIME v1.5.0.

Although all mice received the same DMH treatment in the experimental group, some mice had no tumor formation by the end of our experiment (26 weeks after DMH treatment). We compared the difference of gut microflora between the mice with tumors and those mice without tumors in experimental group. By beta diversity analysis, we found their microbiota were obviously separated (Figure 6). From the genus level, the abundance of Eubacterium was significantly decreased in the tumor mice (*P* = 0.013).We also noticed that Streptococcus, Prevotella and Akkermansia were disappeared in the tumor mice.

### Dynamic detection of the molecular basis of colorectal carcinogenesis

**Figure 7:**
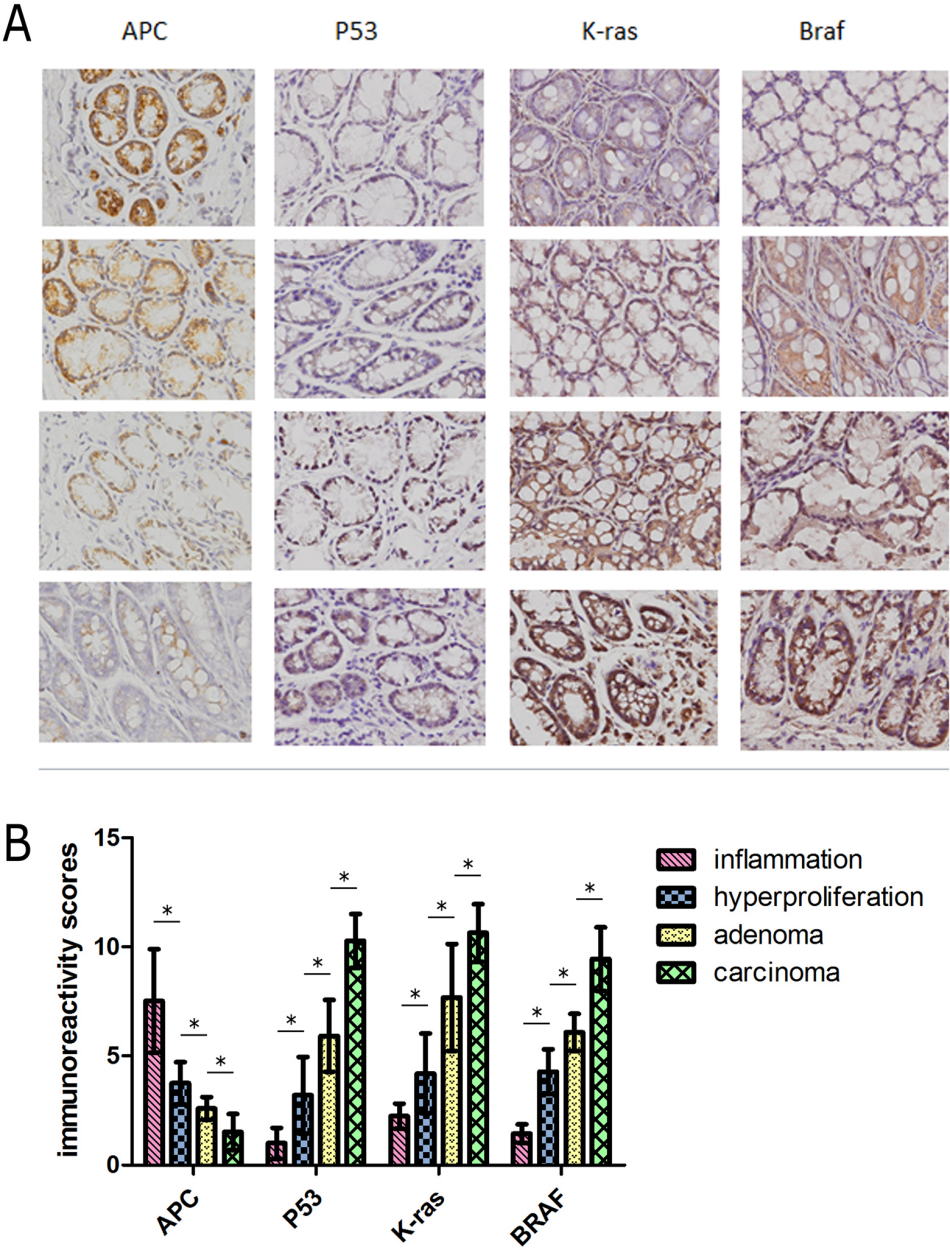
(**A**) Representative staining for four molecules reflecting the pathological process of CRC. The rows represent the each experimental group (*n* = 30). Images were magnified 400×. (**B**) Dynamic changes in expression levels of molecules were semi-quantified with immunoreactivity scores. *: statistically signifcant *P* < 0.05

To detect changes in levels of the molecular factors involved in the ‘adenoma-carcinoma’ sequence, we constructed a time-dependent dynamic alteration map of *APC, P53, K-RAS* and *BRAF* via immunohistochemical staining. *APC* and *P53* are significant factors in tumor formation [18, 19]. The Wnt signaling pathway is regarded as a key event in initiation of colorectal cancer in which *APC* plays an important role. In the normal group, *P53* staining was almost undetectable. However, expression of *P53* was gradually increased in the hyperproliferative, adenoma and carcinoma groups, while *APC* showed the opposite expression pattern. Increased accumulation of *P53* was observed in the villus and cell nucleus (Figure 7A). Additionally, semi-quantification of the expression of these molecules in each group revealed significant variations (*P* < 0.05) (Figure 7B). *K-RAS* and *BRAF* showed a consistent trend of increased expression from the hyperproliferative to adenoma and subsequent carcinoma groups (Figure 2). The observed modifications in expression levels of these molecules indicates dynamic patterns of the ‘adenoma-carcinoma’ sequence of CRC.

## DISCUSSION

Populations of gut microbiota in mice and humans are shared to alarge extent, which are representative not only of the same phyla but also a substantial fraction of common genera, and the similarity increases with sequencing depth [20]. Here, we generated a modified ‘adenoma–carcinoma sequence’ mouse colorectal cancer model induced by 1, 2-Dimethylhydrazine to investigate microbiota community variations during tumor formation progression. The initial step in the activation of DMH is series of oxidative steps that occur in the liver. Its metabolism ultimate activated to electrophilic carcinogens by the action of colon tissue enzymes and bacteria hydrolysis. There is need to be claimed that the half-life period of DMH and its metabolisms was over ten hours, however, from the last injection of DMH to the inflammation group was a week. We speculated that DMH has been completely metabolized in the inflammation group and the followed groups, DMH and its metabolites in the host residues have been negligible. Molecular changes were also examined during the ‘adenoma-carcinoma’ sequence. This animal model study may be useful in exploring the steps leading to human disease. In our study, transition from colon epithelium hyperproliferation through adenoma to adenocarcinoma occurred as a series of separate stages, but was a consecutive process complicated with microbe imbalance. The association between microbiota evolution, molecular changes and tumor development at every stage is therefore the key to understanding disease pathogenesis.

The development of next-generation sequencing technology has facilitated determination of the composition of gut microbiota in detail. In our study, 454 pyrosequencing based on the Roche 454 FLX+ platform was employed to detect the bacterial communities in stools. We observed a dynamic and previously undescribed gut microbiota shift in tumorigenesis. At the early stage of tumor formation, significant differences in *Bacteroidetes* between model and control groups were observed. We detected relative lower abundance of *Firmicutes* and *Proteobacteria* in the intestinal lumen of model mice, and variation of the gut microbiota was a dynamic process. Many of the bacterial species displayed increase-decrease trends, in accordance with the ‘driver-passenger’ model of Tjalsma et al. [7]. *Clostridium lactatifermentans* and *Bacteroides dorei,* characterized as ‘driver’ bacteria, were enriched in the inflammatory and hyperproliferation groups, and showed an obvious decrease with development of tumor. Conversely, *Alistipes finegoldii* increased step-by-step during tumorigenesis, supporting a role as a ‘passenger’ bacterium.

The potential roles of infection and inflammation in the pathogenesis of colorectal cancer cannot be overlooked [21]. The family of *Enterobacteriaceae* also participate in the mechanism of colorectal cancer. Housseau and Mangan [22, 23] showed that the actions of *Enterobacteriaceae* are similar to the prolonged inflammatory response induced by ETBF. *Escherichia coli,* a member of this family, contains the polyketide synthase (pks) genotoxic island encoding a genotoxin known as coli bactin that can induce singlestranded DNA breaks [24, 25]. Subsequent activation of DNA damageinduced signaling pathways, in turn, increases the mutation rate of infected cells [26, 27]. Our results showed significant enrichment of *Enterobacteriaceae* in the model group, which was eventually outcompeted by opportunistic passengers during tumorigenesis.

It is likely that the altered microbial communities in our experiments belong to the same family and perform similar roles. These OTU-related species may be considered biological markers that influence longitudinal microbiota community changes during the development of ‘adenoma-carcinoma’ sequence colorectal cancer.

Recent research indicates a complex link between gut microbiome, immunity, and intestinal tumorigenesis. Mima [28] and colleagues confirmed that *F. nucleatum* is inversely associated with CD3+ T-cell density in colorectal carcinoma tissue. Chronic inflammation and tumorigenesis also probably result in loss of members of the microbial community important for maintaining epithelial health and immune homeostasis, and an increase in some opportunistic pathogens may intensify chronic inflammation and tumorigenesis [16]. *Alistipes finegoldii,* the first bacterium isolated mainly from appendiceal tissue samples in children with acute appendicitis, is bile-resistant and displays positive indole reaction. Annotation of the complete genome sequence by Konstantinos et al. [29] revealed that the genome is mainly associated with cell cycle control, cell division, replication, recombination and repair, intracellular trafficking and secretion, carbohydrate, amino acid, nucleotide, lipid transport and metabolism. A recent human study showed that *Alistipes* are enriched in CRC patients, compared with both healthy and advanced adenoma groups [30]. In our study, the three OTUs (*OTU0151, 0105, 0022*) related to *Alistipes finegoldii* were consistently increased during tumor development. We speculate that due to dysbiosis of gut microbiota and loss of barrier function of colon epithelium, *Alistipes finegoldii* may influence the cell cycle of colon epithelium and ultimately affect cell cycle regulation and leading to epithelial and tumor cell proliferation. Additionally, we noted a dramatic decrease in the family *Porphyromonadaceae* and increase in Rikenellaceae. We hypothesize that *Porphyromonadaceae* bacteria play a protective role and are important mediators of microbiota balance in the murine gut. One potential mechanism underlying this protective activity may be participation in gut metabolism and providing short-chain fatty acids. SCFA have a critical influence on colonic health, although their contribution to human energy requirement is far lower [31, 32]. Recently, the complete genome sequence of the family *Porphyromonadaceae* was reported, shown to contain genes catalyzing the production of volatile fatty acid-encoding proteins and enzymes related to the degradation of complex carbohydrates and proteinaceous compounds. Accordingly, *Porphyromonadaceae* is proposed to be involved in hydrolysis and acidogenesis during anaerobic digestion and biomethanation [33]. It is additionally possible that members of the family Porphyromonadaceae produce anti-inflammatory mediators that modulate the tumor immune microenvironment. Moreover, we speculate that the *Rikenellaceae* family functions as opportunistic pathogens via intensification of inflammation or production of mutagenic toxins.

CRC development can be effectively described by the genetic ‘adenoma–carcinoma sequence’ model originally developed by Fearon and Markowitz [3, 34]. The hypothesis states that loss of genomic stability, such as accumulating genetic and epigenetic mutations, can drive epithelial dysplasia and hyperplasia in the colon, eventually resulting in CRC. Over the course of progression, some ‘driver’ bacteria, such as *enterotoxigenic Bacteroides fragilis* or the *Enterobacteriaceae* family, induce inflammation, dysplasia, hyperplasia and/or genotoxic substances that contribute to the accumulation of mutations during the ‘adenoma–carcinoma sequence’. According to the study of Fearon and Markowitz, mutations start in the *APC* gene, leading to transition to adenoma, ending with a *P53* substitution that triggers carcinoma. With microenvironment alterations during the oncogenic process, driver bacteria are gradually replaced with ‘passengers’ consisting of tumor-foraging opportunistic pathogens, such as *Streptococcus spp*., commensal or probiotic bacteria (e.g. *Coriobacteriaceae*) or other bacteria with a competitive advantage in the tumor niche. These previous studies agree with our findings. *Bacteroidaceae* and *Enterobacteriaceae* showed higher relative abundance in the early stages of tumor formation, and were gradually replaced with *Rikenellaceae*, *Lachnospiraceae*, *Ruminococcaceae* and *Streptococcaceae*. In particular, *Clostridium lactatifermentans* and *Bacteroides dorei*, characterized as ‘driver’ bacteria, were enriched in the inflammatory and hyperproliferation groups, followed by an obviously decrease with tumor development. *Alistipes finegoldii* from three related OTUs increased gradually during the process of tumorigenesis, possibly owing to activity as a ‘passenger’ bacterium. CRC has often been associated with activation of the Wnt/β-catenin pathway. Colitis-associated cancer mutations are reported to affect the Wnt/β-catenin pathway following mutations in the *TP53* and *K-RAS* genes [35].

Sears and Goodwin [36, 37] reported that some bacterial pathogens are directly and specifically involved in promoting CRC. For example, *Enterotoxigenic* Bacteroides fragilis (ETBF) induces spermine oxidase-dependent ROS production and consequent DNA damage. *F. nucleatum* binds E-cadherin on epithelial cells and activates β-catenin signaling, driving epithelial cell proliferation [38]. These bacterial pathogens are thought to participate in tumorigenesis via several common pathways. In particular, STAT3 is broadly implicated in tumorigenesis, inducing suppression of apoptosis and promotion of cell cycle progression [39]. Apart from inflammatory cytokine signaling, activation of oncogenes and inactivation of suppressor genes are steps involved in the initiation of CRC, such as *APC* activation of Wnt signaling, due to an inability to decrease β-catenin oncoprotein [18]. Oncogenic mutations of *RAS* and *BRAF* activate the mitogen-activated protein kinase (MAPK) signaling pathway in 37% and 13% colorectal cancers, respectively [40–44]. RAS mutations, principally *K-RAS,* activate GTPase activity that signals directly to RAF. The *K-RAS* gene encodes a G-protein involved in a constitutively activated signaling pathway that promotes cell survival and apoptotic suppression [45]. Previous studies have reported that *BRAF* mutations occur, even in small polyps [40].

According to Winter et al. [46], dysbiosis of microbiota produces an abundance of potentially harmful microorganisms that induce inflammatory processes. Inflammation caused by gut bacteria is also considered to have an impact on carcinogenesis, leading to damage of intestinal barrier function, bacterial translocation and secretion of cytokines that maintain an inflammatory environment within the tumor [47]. The interactions between microbiota and molecular pathways underlying CRC require further investigation.

To a considerable extent, this work provides mechanistic insights into the interactions between the host and microbes during the process of tumor-promoting evolutionary changes. However, intestinal microecology is an extremely complex and diverse microbial community, and it is unlikely that one mechanism or hypothesis is applicable to all CRC patients. Clearly many questions remain involving whether the microbial community differences found in the present study are cause or consequence of tumor formation. We still need to provide more detailed information that concerning germ-free animals and transplantation of pathogenic bacteria. Further investigation is warranted to confirm the mechanisms underlying dysbiosis, which would aid in the development of effective methods to pre-diagnose CRC.

In summary, by establishing the network involving microbes and cellular molecules throughout the evolutionary progression of CRC tumorigenesis in the ‘adenoma-carcinoma sequence’ mouse colorectal cancer model, we have identified an imbalance in gut microbiota, characterized by the ‘driver-passenger’ model involve in reduction of butyrate-producing bacteria and increase in DNA-damaging bacteria, which leads to enhanced accumulation of mutations. These steps may be significant features of dysbiosis in animal models of CRC.

## MATERIALS AND METHODS

### Animals and reagents

Four-week-old male ICR mice (18–22 g) purchased from Qingdao Laboratory Animal Co. Ltd. (Qingdao, China) were used for study. All animals were maintained in plastic cages (five mice/cage) under conditions of humidity (44 ± 5%), light (12 h light/dark cycle) and temperature (22 ± 2°C), and fed certified standard mice chow and tap water *ad libitum* according to the institutional and National Institutes of Health (NIH) guidelines [48]. 1, 2-Dimethylhydrazine (DMH) was purchased from Beijing J&K Scientific Co. Ltd. DMH solution was prepared freshly before use in 25 mM EDTA/137 mMNaCl carrier (pH 6.4). All animal experiments were approved by the University Committee on the Use and Care of Animals at Qingdao University, and conducted in accordance with the National Institutes of Health guidelines.

### Establishment of the ‘adenoma-carcinoma sequence’ animal model and experimental procedures

We modified the ‘adenoma-carcinoma sequence’ mouse CRC model induced by DMH. For generating the model, DMH induction was performed by administering a large dose (~35–40 mg/kg body weight) and increased number of injections (once a week for10–26 weeks) to ensure a high rate of morbidity [49, 50]. The model was modified to decrease the number and dose of injections. Consequently, morbidity was decreased and a percentage of lesion-free mice identified in each group. ‘Adenoma-carcinoma sequence’ CRC is a multistep process, from normal epithelial to neoplasm in a single crypt to the hyperproliferative stage, followed by adenoma, and finally adenocarcinoma [51, 52]. These steps are distinct but continuous in the morphological context.

After one week of acclimatization, mice were randomly divided into two groups. The model group (*n* = 120) received a subcutaneous (s.c.)injection of DMH solution at a dose of 20 mg/kg body weight once a week for six consecutive weeks while the control group received a subcutaneous injection with the same volume of EDTA/NaCl carrier. On weeks 6, 12, 18 and 26, all mice in the model and control groups were anaesthetized via peritoneal injection of Ketamine (100 mg/kg), and sacrificed via cervical dislocation. The entire colon and rectum was surgically removed and opened longitudinally along the antimesenteric margin. Fresh feces of animals from both the model and control groups were collected after sacrifice, frozen immediately in liquid nitrogen, and stored at −80°C until DNA extraction. Tissues were washed with normal saline for observation, images obtained, and the total number of lesions counted. For histological examination and immunohistochemistry (IHC), tissues were fixed in 10% neutral phosphate-buffered formalin.

### Histological and immunohistochemical examination

IHC was performed using standard protocols [53]. The entire mucosa was examined for macroscopic changes. Portions of the large bowel and tumors in this region were processed for paraffin embedding. Blocks were sectioned at 5 μm intervals, and slides stained with standard hematoxylin and eosin for light microscopic examination. Tissue sections were reviewed independently by two pathologists and re-evaluated by a third examiner in case of ambiguous results. Serial tissue sections 4 mm thick prepared from formalin-fixed and paraffin-embedded tissues were subjected to immunohistochemistry. IHC was completed in accordance with standard protocols using monoclonal antibodies against *P53* (sc-6243, Rabbit, Santa Cruz), *APC* (SAB4200594, Rabbit, Sigma-Aldrich), *K-RAS* (sc-30, mouse, Santa Cruz) and *BRAF* (sc-9002, Rabbit, Santa Cruz).

### DNA extraction, amplification and pyrosequencing

Genomic DNA was extracted from ~ 0.3 g of each sample in duplicate using the FastDNA SPIN Kit for Soil (Qbiogene-MP Biomedicals, Irvine, CA, USA), according to the manufacturer's instructions. The V3-V5 hypervariable region of 16S rDNA genes was amplified using the primers 907F (5′-CCGTCAATTCMTTTGAGTTT-3′) and 338R (3′–ACTCCTACGGGAGGCAGCAG-5′). Each forward primer with a unique 10 bp barcode was used to tag each sample. The amplification mixture contained 25 μL Failsafe Premix F (Epicentre Biotechnologies, Madison, WI, U.S.A.), 0.4 μM each primer, 2.5 U of Ex Taq DNA polymerase (Takara, Dalian, China) and 1–2 μL DNA template in a total volume of 50 μL. PCR reactions were performed at 95°C for 5 min, followed by 25 cycles of 95°C for 30 s, 56°C for 30 s and 72°C for 90 s, and final extension at 72°C for 7 min. All samples were amplified in triplicate, pooled, and purified using the QIAquick PCR purification kit (Qiagen, Valencia, CA, USA). Pyrosequencing was performed on a 454 Genome Sequencer FLX platform (Roche) using the GS FLX Titanium XLR70 sequencing kit according to Roche Sequencing Method (Manual GS FLX Titanium Series October 2009 Edition). All DNA extraction, amplification and pyrosequencing procedures were operated by a professional at BGI (Shenzhen, China).

### Bioinformatics analysis

Raw pyrosequencing data were processed using Mothur (Version 1.31.2, http://www.mothur.org/) to obtain unique reads. Ribosomal Database Project (RDP) Classifer v.2.2 was used to taxonomically classify OTU representative sequences in the following databases: Greengene V201305; RDP (Release9 201203).

### Statistical analysis

Diversity indices, including the nonparametric richness estimator observed_species, Chao, Shannon, and Simpson index, were used to calculated the value of the sample Alpha diversity with Mothur (Version 1.31.2) and dilution curve generated using the Rprogram (Version 2.15.3). The Mann–Whitney test was applied to evaluate differences in the bacterial populations of each group. β-diversity and UniFrac analyses were carried out using QIIME v1.5.0. Significant difference analyses were calculated using Metastats (http://metastats.cbcb.umd.edu/), and *P* values corrected in R (Version 2.15.3). Data were considered statistically significant at *P* < 0.05. The Kruskal-Wallis rank sum test was employed to evaluate the differences between each group. Spearman's rank correlation was used to assess the relationship between variables. Data analyses were performed in SPSS version 19.0.

## Abbreviations

CRC: colorectal cancer
DMH: 12-Dimethylhydrazine
APC: adenomatous polyposis coli
OTU: operational taxonomic units
ROS: reactive oxygen species
SCFA: short-chain fatty acids
